# Inhibition and Updating, but Not Switching, Predict Developmental Dyslexia and Individual Variation in Reading Ability

**DOI:** 10.3389/fpsyg.2018.00795

**Published:** 2018-05-28

**Authors:** Caoilainn Doyle, Alan F. Smeaton, Richard A. P. Roche, Lorraine Boran

**Affiliations:** ^1^School of Nursing and Human Sciences, Dublin City University, Dublin, Ireland; ^2^Insight Centre for Data Analytics, Dublin City University, Dublin, Ireland; ^3^Department of Psychology, Maynooth University, Kildare, Ireland

**Keywords:** dyslexia, executive function, inhibition, updating, processing speed, reading

## Abstract

To elucidate the core executive function profile (strengths and weaknesses in inhibition, updating, and switching) associated with dyslexia, this study explored executive function in 27 children with dyslexia and 29 age matched controls using sensitive z-mean measures of each ability and controlled for individual differences in processing speed. This study found that developmental dyslexia is associated with inhibition and updating, but not switching impairments, at the error z-mean composite level, whilst controlling for processing speed. Inhibition and updating (but not switching) error composites predicted both dyslexia likelihood and reading ability across the full range of variation from typical to atypical. The predictive relationships were such that those with poorer performance on inhibition and updating measures were significantly more likely to have a diagnosis of developmental dyslexia and also demonstrate poorer reading ability. These findings suggest that inhibition and updating abilities are associated with developmental dyslexia and predict reading ability. Future studies should explore executive function training as an intervention for children with dyslexia as core executive functions appear to be modifiable with training and may transfer to improved reading ability.

## Introduction

Although developmental dyslexia is a neurodevelopmental disorder characterised by reading (such as accuracy and speed problems) and phonological difficulties (awareness and implementation of sound structure of language), despite adequate instruction and intellectual ability (World Health Organization, 1992; American Psychiatric Association, 1994, 2013). Executive function impairments are frequently observed.

Executive function is an umbrella term for a range of high-level cognitive processes associated with frontal regions of the brain which subserve goal-directed behaviour. Executive function is what enables us to represent and manipulate goal-related information in a highly active state, focus our attention in the face of distraction, update goal relevant information in working memory, rapidly adapt to changing demands within our environment and plan our actions accordingly. Although in agreement on the importance of executive function for directing behaviour, most theories define and segment the elusive concept of executive function differently (see Jurado and Rosselli, 2007 for a more comprehensive review of executive function and associated theories). Baddeley and Hitch (1974) proposed that working memory is comprised of two domain specific storage components (visuo-spatial sketchpad and phonological loop) and one domain general control component (central executive). Within their model, the central executive is defined as the component responsible for the manipulation of information, focusing attention on relevant and inhibiting irrelevant stimuli, regulating performance across multi-tasking conditions, and planning behavioural sequences (Baddeley and Hitch, 1974). The Supervisory Attentional System (SAS) conceptualised by Norman and and Shallice (1986) is an attentional control mechanism necessary for the initiation of effortful goal-directed behaviours (requiring planning, error monitoring and resisting) as opposed to automatic effortless behaviours. The SAS selection and control of actions depends upon contention scheduling, a process involving anatagonistic activation and inhibition of action schemas (Norman and and Shallice, 1986). Within both models, executive function is labelled as a unitary component responsible for multiple sub-functions. Executive function is also often measured with complex tasks such as Tower of London, Wisconsin Card Sort Task, and complex span tasks, which tap multiple sub-functions together and are sensitive for detecting profuse executive dysfunction in frontal lesion patients.

More recent work on the latent factor structure of executive function in typical samples indicates that executive function is comprised of a set of core related (through the common executive function: inhibition) and distinct (updating specific and switching specific) processes which contribute differentially to complex tasks and may be antagonistically related (trade-offs between inhibition and switching specific; Miyake et al., 2000; Miyake and Friedman, 2012; Snyder et al., 2015). Complex executive function tasks therefore lack the specificity to detect the fine-grained core executive processes of inhibition, updating and switching (Miyake et al., 2000; Snyder et al., 2015), particularly in conditions which are associated with more subtle impairments rather than the severe executive impairments observed in lesion patients (Snyder et al., 2015). This is not to say that complex executive processes such as planning, decision making, problem solving, and verbal fluency are not “executive.” Within Diamond's (2013, p.136) model of executive function, these complex processes are classified as higher-order executive processes which are “built” from the core executive processes of inhibition, working memory and switching. As such, there is a value in establishing the core executive profile associated with a condition before we begin to consider how higher-order executive processes are impacted. Miyake and Friedman (2012) provide a useful framework for exploring and measuring the core executive functions of inhibition, updating and switching. Inhibition is defined as the ability to override inappropriate responses, regulate appropriate behaviour and control attention by focusing on relevant information and filtering out distracting information; updating is the ability to hold and continuously update information in working memory from moment to moment; and switching is the ability to rapidly adapt to changing task demands (Miyake et al., 2000; Miyake and Friedman, 2012; Diamond, 2013).

The core executive functions may contribute to typical reading ability in many ways. Efficient reading requires the coordination of multiple processes such as focusing of attention on visual information, decoding visual information into speech sounds, maintaining, and updating speech sounds in working memory, combining speech sounds, matching combinations of speech sounds with stored words, deriving semantic meaning for comprehension, and moving onto the next word to start this process again. Beyond efficient functioning of each stage separately, these processes need to be carried out rapidly, sometimes in parallel and efficient switching between each stage is required. Inhibition may contribute to reading ability by focusing attention on relevant visual information, ignoring irrelevant information, maintaining speech sounds active and protected from interference in working memory while other stages are completed. In addition, children are often faced with reading in somewhat noisy and distracting environments such as the classroom, where additional demands are placed on inhibition to filter out distracting information. Updating may contribute to reading ability by holding and updating speech sounds in working memory during ongoing decoding of text and combining old speech sounds with new speech sounds to enable full word reading and comprehension. Switching processes may also contribute to reading, and given that multiple processes are involved in reading, switching abilities may support rapid alteration between different stages in the reading process which may support reading speed.

There is evidence for genetic linkages between executive function and reading development, Kegel and Bus (2013) found that genes important for the development of dopamine receptors in pre-frontal brain areas (DRD4) predict the acquisition of alphabetic skills important for reading from kindergarten to first grade, with executive function mediating this relationship. Some studies have also found that dyslexia is associated with underactivity of parietal and prefrontal areas important for executive function during an updating task (Beneventi et al., 2010), and abnormal neurophysiological markers of executive functioning during a range of executive tasks (Beneventi et al., 2010; Liotti et al., 2010; Van De Voorde et al., 2010; Horowitz-Kraus, 2014).

Despite evidence of genetic linkages between executive function and reading and reduced activity in brain areas supporting executive function in dyslexia, thus far, the exact core executive function profile (strengths and impairments in inhibition, updating, and switching; Miyake and Friedman, 2012; Friedman and Miyake, 2016) associated with dyslexia is unclear. Although some studies report that dyslexia is not associated with executive function impairments (Bental and Tirosh, 2007; Smith-Spark and Fisk, 2007; Peng et al., 2013; Bexkens et al., 2014), the majority of the literature thus far point to impairments (Nydén et al., 1999; Helland and Asbjørnsen, 2000; Willcutt et al., 2001, 2005; Brosnan et al., 2002; van der Sluis et al., 2007; Beneventi et al., 2010; Menghini et al., 2010; Poljac et al., 2010; Moura et al., 2016; see Table 1). However, there are conflicting findings regarding exactly which executive functions are compromised in dyslexia. A number of studies report inhibition impairments in dyslexia (Willcutt et al., 2001, 2005; Brosnan et al., 2002; De Lima et al., 2012; Booth et al., 2014; Proulx and Elmasry, 2014), while others do not (Reiter et al., 2005; Bental and Tirosh, 2007; Marzocchi et al., 2008; Schmid et al., 2011; Bexkens et al., 2014). A number of studies report updating (working memory) impairments in dyslexia (Brosnan et al., 2002; Rucklidge and Tannock, 2002; McGee et al., 2004; Willcutt et al., 2005; Bental and Tirosh, 2007; Smith-Spark and Fisk, 2007), while others do not (Willcutt et al., 2005; Marzocchi et al., 2008; Peng et al., 2013). Likewise, a number of studies report switching impairments in dyslexia (Helland and Asbjørnsen, 2000; Poljac et al., 2010; De Lima et al., 2012), while others do not (Reiter et al., 2005; Bental and Tirosh, 2007; Marzocchi et al., 2008; Tiffin-Richards et al., 2008; Menghini et al., 2010). A meta-analysis conducted by Booth et al. (2010) indicates that dyslexia is associated with executive dysfunction (Hedges g = 0.57), however, effect sizes vary across tasks due to underlying task demands, these task impurity issues make it difficult to conclude on the exact profile of core executive functions in dyslexia.

**Table 1:** Summarising characteristics of previous EF profiling studies in dyslexia.

**Authors**	**Sample (N)**	**Age**	**Grouping**	**ADHD: Screened**	**Cont. speed**	**Profiling approach**	**Measure**	**Findings**
Pennington et al., 1993	D:15 C:23	D:9.1 C:8.8	CD	ST	–	UA	EF comp. (TOH, MFF, CPT)	–
Nydén et al., 1999	D:10 C:10	D:10.1 C:10.1	CD	ST	–	MUP	GNG, WCST	↓D: GNG
Helland and Asbjørnsen, 2000	D:43 C:20	D:12.67 C: 12.11	CD	NH	–	MUP	Stroop, WCST	↓D: Stroop, WCST
Palmer, 2000	D: 16 C:16	D:14 C:14	CD	–	–	SM	WCST	↓D: WCST
Willcutt et al., 2001	D:93 C:121	D:10.4 C:10.7	RAST (SD 1.65)	ST	–	MUP	WCST, SST, TMT, Stroop	↓D: WCST, TMT, SST, Stroop
Brosnan et al., 2002	D: 30 C:30	D:14 C:13.8	CD	NH	–	SM	GEFT	↓D: GEFT
Jeffries and Everatt, 2004	D:21 C: 40	D:10.8 C:11.07	CD	–	–	SM	Stroop	
Reiter et al., 2005	D:42 C:42	D: 10.8 C:10.6	CD	NH	–	MUP	FT, GNG, Stroop, TOH, WCST, TMT	↓D: FT, Stroop, TOL
Willcutt et al., 2005	D:109 C:151	D:11 C:11.5	D: RAST (SD 1.75)	ST	–	MUP	SST, CPT, WCST, TMT	↓D: SST, CPT↓
Smith-Spark and Fisk, 2007	D:22 C:22	D:20.59 C:20.82	CD	–	–	SM	CU, SU	–
Bental and Tirosh, 2007	D:17 C:23	D:9.96 C:9.75	CD	ST	–	MUP	MFF, PM, WCST	–
Tiffin-Richards et al., 2008	D:20 C:19	D:11 C:11.7	CD	ST	–	SM	WCST	–
de Jong et al., 2009	D:41 C:26	D:10.1 C:9.31	CD	ST	–	SM	SST	↓D: SST
Marzocchi et al., 2008	D:22 C:25	D:9.43 C:9.72	CD	ST	–	MUP	OW	↓D: OW
Menghini et al., 2010	D:60 C:65	D:11.43 C:11.94	RAST (2 SD)	NH	–	MUP	FT, WCST	↓D: FT
Kapoula et al., 2010	D:10 C:14	D:15.1 C: 14.3	RAST (2SD)	NH	–	SM	Stroop	↓D: Stroop
Poljac et al., 2010	D:25 C:27	D:15.4 C:15.2	CD	–		SM	MT	↓D: MT
Beneventi et al., 2010	D:11 C:13	D:13.2 C:13.5	CD	NH	–	SM	P2-back	↓D: P2-back
Gooch et al., 2011	D:17 C:42	D:10.69 C:10.27	CD	NH	–	SM	SST	–
Schmid et al., 2011	D:20 C:16	D:9.7 C:9.3	RAST	NH	–	SM	SST	↓D: SST
Beidas et al., 2013	D:34 C:35	D:25.32 C:25.02	CD	NH	–	UA	EF Comp. (TOL, WCST, Stroop)	↓D: EF comp.
De Lima et al., 2012	D:20 C:20	D: 9.7 C:9.05	CD	NH	–	MUP	TMT, Stroop, TOL, WCST	↓D: TMT, Stroop ↑WCST
De Weerdt et al., 2013	D:17 C:45	D:9.96 C:10.08	CD	NH	–	SM	AN-GNG, Pi-GNG	↓D: AN-GNG
Peng et al., 2013	D:22 C:31	D:11.09 C:10.99	RAST (25th perc.)	NH	Yes	MUP	Stroop, Num-Stroop, W2-back, N2-back	–
Bexkens et al., 2014	D:28 C:31	D:10.11 C:11.2	RAST (1 SD)	NH	–	SP	SST, Sim. T	–
Varvara et al., 2014	D:60 C:65	D:11.4 C:11.9	CD	ST	–	MUP	WCST, FT	↓D: FT
Moura et al., 2015	D:50 C:50	D:9.8 C:9.82	CD	NH	–	MUP	TMT, TOL, FT	↓D: TMT, FT
Wang and Yang, 2015	D:37 C:37	D:10.1 C:10	RAST	–	–	SP	Cog inhib comp. (Stroop, GEFT), Behav. Inhib comp (GNG, SST)	↓D: Cog inhib comp
Moura et al., 2016	D:32 C:34	D:9.00 C: 9.03	CD	NH	–	MUP	TMT, FT	↓D: TMT

*D, dyslexia; C, control; CD, clinical diagnosis; RAST, researcher administered standardised test; SD, standard deviation; ST, standardised tool; NH, no history; UA, unified ability; MUP, multiple unrelated processes; SM, single measure; SP, single process; EF, executive function; Comp, composite score; TOH/L, Tower of Hanoi/London; MFF, Matching Familiar Figures; CPT, Continuous Performance Test; GNG, Go No-Go; WCST, Wisconsin Card Sort Test, SST, Stop Signal Task; Stroop, Stroop Task; TMT, Trail Making Task; GEFT, Group Embedded Figures Task; FT, Fluency Task; CU, Consonant Updating; SU, Spatial Updating; PM, Porteus Maze; OW, Opposite Worlds (TEACH); MT, Matching Switch Task; P2-back, phoneme 2-back, AN-Alphanumeric; Pi, Pic; Num, Number; W2-back, Word 2-back Task; N2-back, Number 2-back Task; Sim. T, Simon Task; Cog, Cognitive; inhib, inhibition; Behav, Behavioural*.

Conflicting findings also emerge across studies exploring the predictive ability of executive function for dyslexia likelihood and for explaining variance in reading abilities. For instance, Booth et al. (2014) found that inhibition and updating combined predict dyslexia likelihood, while Moura et al. (2015) found that switching alone predicts dyslexia likelihood. Yet others report that executive function does not predict dyslexia likelihood (Willcutt et al., 2010). In typical samples, executive function appears to explain variance in reading skills, however, it is unclear which core executive functions (inhibition, updating, and switching) are predictive of reading. Some studies find that working memory updating predicts word reading ability (Christopher et al., 2012), while others argue that switching predicts word reading ability (Cartwright, 2012). There is also evidence that a combination of executive functions predict word reading ability, yet studies also differ regarding *which combination of executive functions* predict reading. For instance, van der Sluis et al. (2007) found that updating and switching combined are predictive of word reading ability, while other studies suggest that inhibition and updating combined are predictive of word reading ability (Welsh et al., 2010; Arrington et al., 2014). Some authors have also found that a combination of updating and processing resources such as speed predict reading ability in typically developing children (Christopher et al., 2012).

In atypical reading samples (dyslexia), it is unclear whether executive function predicts reading problems, as some studies find a predictive relationship, while others do not. Those reporting that executive function is implicated in word reading problems find that a different combination of executive functions explain variability. For instance, some studies find that impaired inhibition and updating combined predict reading problems in dyslexia (Wang and Yang, 2015), while others find that impaired inhibition and switching combined predict reading problems in dyslexia (Altemeier et al., 2008). However, some studies do not report a predictive relationship between executive function and reading problems. Instead, a combination of working memory capacity, processing speed and phonological abilities predict reading problems (McGrath et al., 2011) or processing speed and phonological abilities predict reading problems (Peterson et al., 2016).

Inconsistencies in the type of executive functions impaired in dyslexia and of clinical relevance for predicting dyslexia likelihood and reading ability, may be due to differences in sample characteristics/criteria, theoretically informed profiling approach, measurement tools and systematic control of confounding variables across studies (see Tables 1, 2). These issues make it difficult to infer the exact core executive function profile associated with dyslexia and whether variability in core executive functions are of clinical relevance for predicting dyslexia likelihood and variance in reading ability.

**Table 2:** Summarising characteristics of executive function predictive studies of reading and dyslexia.

**Authors**	**Sample (N)**	**Age**	**Grouping**	**ADHD: Screened**	**Cont. speed**	**Profiling approach**	**Measure**	**Findings**
Booth et al., 2014	D:21 CA:21 RA:21 A tracked: 9 D with A risk	D:10.7 CA: 10.6 RA:7.5	RAST (< 15th perc.)	–	–	MUP	Inhib. Comp (EA-CAS, ND-CAS) BDS, MD-SCPS	M1: Inhib. Comp & BDS distinguished D from CA & D from RA. M2: Inhib. Comp & MD-SCPS distinguished D from CA & D from RA. M2 outperformed M1
Moura et al., 2015	D:50 C:50	D:9.8 C:9.82	CD & RAST (15th perc.)	NH	–	MUP	TMT, TOL, FT	TMT-B distinguished D from C
Christopher et al., 2012	Y-C: 266 O-C: 217 D + A tracked: 128 D, 98 A in samples above.	Y-C: 8-10 O-C: 11-16	–	–	YES	LA	WM, LF (DS, SS, CS) Inhib. LF (CPT, SST) CPST, RAN	WM & CPST predict reading in Y-C & O-C
van der Sluis et al., 2007	C:172	C:10.67	–	–	–	LA	Naming LF (QI, OI, Stroop, NSI, OS, SS, PS, MT, KT, LM, DM), UP, LF (KT, LM, DM), SW. LF (OS, SS, PS, TMT)	Naming, UP. & SW. predict reading
Arrington et al., 2014	C: 1134	C:11.35-17.6	–	–	–	MUP	NRS, SST, VPI	WM (NRS) and Inhib. (SST) predict word reading
Welsh et al., 2010	C:164	C: 4.49 (T1), 5.59 (T2), 6.59 (T3)	–	–	–	UA	EF comp. (BWS, PTT, DMCST)	EF at 4.49 predicts reading at 6.59. EF predicted growth in emergent literacy
Wang and Yang, 2015	D:37 C:37	D:10.1 C:10	RAST	–	–	SP	Cog inhib comp. (Stroop, GEFT) Behav. Inhib comp (GNG, SST)	Cog inhib. predicts reading in D & C
Altemeier et al., 2008	S1 1G-4GC: 128, 3G-6GC: 113 S2: D: 122, 5GC: 106 A tracked: 4 D with A.	S1: NR S2: D: 11y 6m, 5GC: 10 y 7m	RAST (R 2.8SD, S 1.6 SD)	–	–	MUP	CWI, CWI-SS, RAS	S1: CWI & RAS predict reading across 1G-4G, strongest in 3G. S1: EF development 1G-4G predicts reading in 4G. S2: CWI & RAS predict reading in D and C.

*D, dyslexia; A, ADHD C, control; CA, chronological age controls; RA, reading age controls; Y-C, young controls; O-C, old controls; 1G-4G C, 1st- 4th grade controls; 3G-6G C, 3rd-6th grade controls; 5GC, 5th grade controls; S1, study 1; S2, study 2; NR, not reported; T1, time 1; T2, time 2; T3, time 3; CD, clinical diagnosis; RAST, researcher administered standardised test; SD, standard deviation; Perc, percentile; ST, Standardised Tool; NH, no history; UA, unified ability; MUP, multiple unrelated processes; SM, single measure; SP, single process; LA, latent analysis; EF, Executive function; Comp, composite score; Inhib, inhibition; EA-CAS, Expressive Attention Subtest of Cognitive Assessment System; ND-CAS, Number Detect of Cognitive Assessment System; BDS, Backward Digit Span; MD-SCPS, Mapping and Direction Subtest from Swanson Cognitive Processing Test; M1, model 1; M2, model 2; TMT, Trail Making Task; TOH/L, Tower of Hanoi/London; FT, Fluency Task; WM, working memory; LF, latent factor; DS, Digit Span; CS, Counting Span; SS, Sentence Span; CPT, Continuous Performance Test; SST, Stop Signal Task; CPST, Colorado Perceptual Speed Test; RAN, Rapid Automatized Naming; UP, updating; SW, Switching; QI, Quantity Inhibition; OI, Object Inhibition; Stroop, Stroop Task; NSI, Number Size Inhibition; KTT, Keep Track Task; LM, Letter Memory; DM, Digit Memory; OS, Objects Shifting; SS, symbol switching; PS, place shifting; NRS, Numbers Reversed Subtest; VPI, Verbal Proactive Interference; BWS, Backward Span; PTT, Peg Tapping Task; DMCST, Dimensional Card Sort Task; Cog, Cognitive; Behav, Behavioural; CWI, Colour Word Interference from D-KEFS; CWI-SS, Colour Word Interference Switch Score from D-KEFS; RAS, Rapid Automatic Switching*.

Across executive function profiling and predictive studies there is a discrepancy between how dyslexia is classified within the sample (see Tables 1, 2). Some studies include only participants with a clinical diagnosis of dyslexia given by a clinical/educational psychologist and based on DSM criteria (de Jong et al., 2009; Gooch et al., 2011; Varvara et al., 2014; Moura et al., 2015, 2016), while others use researcher-administered standardised tools to classify dyslexia, which vary in terms of cut-off points for classification (Altemeier et al., 2008; Peng et al., 2013; Bexkens et al., 2014; Booth et al., 2014). Studies also differ with regard to method for screening co-occurring ADHD or potentially undiagnosed ADHD from the dyslexia sample, although some studies implement a standardised tool to screen ADHD from the dyslexia sample (Pennington et al., 1993; Willcutt et al., 2001, 2005; Marzocchi et al., 2008; Tiffin-Richards et al., 2008; de Jong et al., 2009; Varvara et al., 2014), the majority just require no history of a diagnosis or report no method of tracking/screening ADHD from the dyslexia alone sample, or track ADHD but do not screen it from the sample. Not screening ADHD from the dyslexia sample is problematic as these conditions frequently co-occur (Willcutt and Pennington, 2000), and ADHD is associated with executive function impairments (Barkley, 1997). This makes it difficult to determine if executive function impairments are associated with dyslexia alone or manifest due to the presence of elevated ADHD within the sample.

Executive function profiling and predictive studies in dyslexia also differ in terms of approach to measuring executive function (see Tables 1, 2). A number of studies view executive function as a unitary construct (employing complex measures such as Wisconsin Card Sort Task or unitary executive function composites; Pennington et al., 1993; Welsh et al., 2010; Beidas et al., 2013; Moura et al., 2015), whilst others view it as multiple but separate abilities (employing multiple complex measures such as planning, switching, inhibition, interference control, and verbal fluency) (Willcutt et al., 2001, 2005; Altemeier et al., 2008; Menghini et al., 2010; De Lima et al., 2012; Arrington et al., 2014; Booth et al., 2014; Moura et al., 2015, 2016), or look at separate processes in isolation with single tasks or composite scores (Beneventi et al., 2010; Poljac et al., 2010; Schmid et al., 2011; Wang and Yang, 2015). Extensive research carried out on the 3-factor model of executive function suggests that it is comprised of three core related (inhibition-common executive function) but separable abilities (updating and switching) which are most sensitively measured at the latent level with multiple tasks (Miyake and Friedman, 2012). The 3-factor structure of executive function has been found in childhood (Lehto et al., 2003) and adulthood (Miyake and Friedman, 2012), However, Huizinga et al. (2006) found evidence of a 2-factor rather than a 3-factor model across development (7–21 years) with latent factors of updating and switching, but not inhibition emerging. All of the variance of inhibition was not captured by updating and switching, rather inhibition tasks were not treated as a single factor due to low and opposing correlations, therefore they were included as manifest task-level factors in the model which best fit the data (Huizinga et al., 2006). Most executive function profiling and predictive studies do not measure executive function in such a way that they can elucidate the core profile of executive functions associated with dyslexia.

Previous approaches to profiling executive function in dyslexia and modelling its predictive ability for dyslexia likelihood and variance in reading ability are also problematic due to task impurity issues (see Tables 1, 2). Complex tasks are poor profiling tools for detecting fine grained impairments in core executive functions, they lack specificity in detecting key underlying impairments, as performance is driven by a range of core executive functions (inhibition, updating, and switching) and non-executive processes (e.g., learning from feedback in WCST; Miyake et al., 2000; Miyake and Friedman, 2012; Snyder et al., 2015). Viewing executive function as a number of separate unrelated abilities or looking at single processes in isolation does not address how these abilities are facilitated by a number of core underlying processes which are both related (through the common factor: inhibition) and unique (updating and switching) (Miyake and Friedman, 2012). In addition, use of complex or higher-order executive tasks cannot assess potential trade-offs between executive functions (Goschke, 2000; Gruber and Goschke, 2004; Miyake and Friedman, 2012; Blackwell et al., 2014; Snyder et al., 2015; Friedman and Miyake, 2016). For instance, trade-offs have been observed between inhibition and switching due to the incompatibility of each demand (Goschke, 2000; Gruber and Goschke, 2004; Blackwell et al., 2014). Inhibition facilitates increased focus by filtering irrelevant information/distractions in a top-down manner, whilst switching requires a degree of distraction to aid in considering alternative options in order to flexibly adapt to changing task demands (Gruber and Goschke, 2004). Although some authors are exploring more sensitive measurement of executive function by employing latent constructs (van der Sluis et al., 2007; Christopher et al., 2012). The majority of predictive and profiling studies in dyslexia do not employ “pure” measures of core executive functions and this necessarily limits the fine-grained understanding of how the common and specific aspects of executive function are clinically relevant for predicting dyslexia likelihood and explaining variance in reading ability.

A confounding factor in determining whether executive function is impaired in dyslexia and of clinical relevance, is processing speed. Processing speed is an index of the speed of cognitive processing and is considered a general mechanism underpinning performance on a wide range of cognitive tasks, as constrained speed of processing results in poor performance on time limited cognitive tasks (Salthouse, 1996). Similar to executive function, processing speed efficiency increases from childhood into adulthood and reduced efficiency is observed in later adulthood (Kail, 1991; Salthouse, 1996). Processing speed has been found to mediate the age-related changes in inhibition, working memory, and switching from childhood to adulthood (Span et al., 2004), suggesting that it is responsible for developmental changes in executive function. In addition, processing speed has been shown to explain variance in inhibition and switching, but not updating, at the individual task level (Huizinga et al., 2006). Previous research suggests that dyslexia is associated with processing speed impairments compared to control participants and that processing speed is predictive of reading ability (Willcutt et al., 2005, 2010; Shanahan et al., 2006; McGrath et al., 2011; Peterson et al., 2016). Peng et al. (2013) found updating and inhibition impairments in dyslexia, yet when they controlled for general processing speed impairments, updating and inhibition impairments no longer reached significance. This is problematic because poor performance on executive function tasks in dyslexia could be a consequence of impaired processing speed. Not controlling for processing speed then could result in false positive findings of executive impairments, which are reflective of a general slowness as opposed to an executive impairment *per se*.

Although we highlight the importance of controlling for processing speed when exploring whether executive function is impaired in dyslexia and of clinical relevance, this is not to say that processing speed is more important to account for than phonological processes in a predictive model of reading. Phonological impairments are consistently found in dyslexia (Swan and Goswami, 1997; Wimmer et al., 1998), and predict future reading ability (Mann, 1993). However, the focus of the present study is not to establish the predictive nature of executive function for reading ability beyond the contributions of phonological processing or processing speed. Rather, the focus of the present study is to establish the fine-grained profile of executive functions associated with dyslexia and which specific aspects of executive function support reading ability while controlling for the confounding influence of processing speed on executive function performance.

Most executive function profiling and predictive studies do not measure executive function in such a way that they can elucidate the core profile of executive functions associated with dyslexia while accounting for individual differences in processing speed. To address this, our study aims to profile and explore the predictive ability of core executive functions in dyslexia using Miyake and Friedman's (2012) 3-factor model and to employ sensitive measures of each construct whilst controlling for individual differences in processing speed. Tasks were deemed sensitive measures if they: (1) demonstrate significant loadings onto core executive function constructs within previous latent variable analyses studies; and (2) are underpinned by frontal brain activation. Within this study, multiple measures are employed for each executive construct (inhibition, updating, and switching) with different types of content (e.g. picture, phoneme, and alpha-numeric). Following from the work of Beneventi et al. (2010) which found phonemic updating impairments in dyslexia, these tasks were also carefully selected to allow for an exploration of phoneme specific vs. general executive processing in dyslexia. However, a consideration of processing constraints imposed by phonemic content is beyond the scope of this paper. Although, latent variable analysis is considered the most sensitive approach to measure core executive functions (Miyake and Friedman, 2012), it could not be conducted in this study due to sample size constraints. Executive function z-mean composite scores were created for each construct which provide cleaner measures by filtering out any non-executive noise when sample size is constrained (Snyder et al., 2015). This study will include a homogenous sample of participants with a clinical diagnosis of dyslexia and screen for elevated ADHD using the combined ADHD subscale of the Child Behaviour Checklist (Achenbach and Rescorla, 2001). Children scoring in the pre-clinical/clinical range on the Child Behaviour Checklist for their age and gender will be screened from the dyslexia sample. By remedying some of the issues associated with executive function measurement in dyslexia, this study may shed light on the core executive function profile associated with dyslexia and whether this is clinically relevant for variability in reading.

Overall, there is difficulty in determining the core executive function profile of dyslexia and whether core executive functions are clinically relevant for predicting dyslexia likelihood and variance in reading ability. By using z-mean measures of each executive construct, this study aims to establish the core executive function profile (strengths/impairments in inhibition, updating, and switching) associated with dyslexia and determine which core executive functions are predictive of dyslexia likelihood and variance in reading ability while controlling for individual differences in processing speed. Exploring executive function in dyslexia using the 3-factor structure may also elucidate strengths and impairments, as well as potential trade-offs between executive functions which often manifest between inhibition and switching due to incompatibility of each demand (Goschke, 2000; Gruber and Goschke, 2004; Blackwell et al., 2014), thus allowing for the development of a more sensitive and specific executive function profile of dyslexia which cannot be captured by previous profiling approaches.

## Methods and materials

### Participants

Fifty-six participants aged 10–12 years were recruited to take part in this study: 27 participants (13 female, 14 male; mean age: 10.78 years) with developmental dyslexia, and 29 participants (female:12, male: 17; mean age:10.93) with no clinical diagnosis served as a control group. Dyslexia diagnosis was confirmed with a copy of the psychological assessment report conducted by a clinical or educational psychologist. Two participants in the dyslexia group did not have a formal diagnosis of dyslexia but were enrolled on a dyslexia support workshop at their primary school. Initially 31 participants with dyslexia were recruited, however 4 were removed from the analysis due to scoring in the clinical range on the ADHD scale of the Child Behaviour Checklist (Achenbach and Rescorla, 2001). All participants were monolingual English speakers with normal or corrected vision and hearing. Participants had no additional diagnosis of a psychological or neurodevelopmental condition. Informed consent and assent were obtained from participating parents and children in written form. Ethical approval for this research project was granted by Dublin City University's Research Ethics Committee (DCUREC/2014/167) in accordance with the declaration of Helsinki. Participants were recruited through the Dyslexia Association of Ireland and primary schools in Ireland.

### Procedure

The research study was carried out in the psychology laboratories in the School of Nursing and Human Sciences at Dublin City University. All participants were assessed individually in the presence of a parent or guardian. The testing session took ~2 h to complete and a break was taken half way through. During the testing session children completed a battery of neuro-cognitive (executive function), reading and processing speed measures. The order of tasks was counterbalanced for each participant to control for fatigue effects. All neuro-cognitive measures were created with E-Prime Software and responses were recorded on a Cedrus RB-50 response pad.

### Measures

#### Processing speed

Participants completed a computerized version of the coding task (Wechsler, 2003) as a measure of processing speed. On screen participants viewed a row of letters with a row of numbers directly underneath while a letter was presented centrally. Participants were tasked with searching for the centrally presented letter on the letter row and pressing the number on the keypad which was directly underneath the letter. This task consisted of 30 trials and a practice block of 10 trials where feedback was given. The dependent measure in this task is the number of trials correctly completed after 30 s. Latent analyses of the coding task reveal that it loads highly onto a general processing speed factor (Keith et al., 2006; Watkins et al., 2006; Bodin et al., 2009). Although some authors find that this task is correlated with inhibition and predicts variance in working memory (Cepeda et al., 2013). Confirmatory factor analytic studies suggest that this task has higher loadings on a processing speed than a working memory factor (Watkins et al., 2006; Bodin et al., 2009). Watkins et al. (2006) found that the loadings of the coding task on a processing speed factor (0.70) far out-weighed its loadings on a working memory factor (−0.04).

#### Inhibition measures

##### Stroop task

Participants completed the Stroop Task (Balota et al., 2010) as a measure of response inhibition. In this task participants were presented with four colour words (red, blue, green, yellow) and four non-colour words (poor, deep, legal, bad) which were presented on screen in varying ink colours (red, blue, green, yellow). In the first block (colour naming) participants had to press the button on the response pad corresponding to the ink colour of the word. In the second block (word naming) participants had to press the button on the response pad corresponding to the meaning of the word (e.g., press red for word red only). Practice blocks were given before each experimental block which consisted of 16 trials. Experimental blocks consisted of 104 trials. Stimuli appeared on screen for 5,000 ms with an inter-stimulus fixation of 500 ms. Stroop interference effect scores for errors and reaction time were calculated by subtracting reaction time/errors on congruent trials from reaction time/errors on incongruent trials. The Stroop task significantly loads onto an inhibition latent variable (Miyake et al., 2000; Friedman and Miyake, 2004), and is underpinned by frontal brain activation (Bench et al., 1993; Collette et al., 2005).

##### Picture Go No-Go task

Participants completed the picture Go No-Go task as a measure of inhibition. This task was an adapted version of the Go No-Go task (Brocki and Bohlin, 2004; McAuley and White, 2011) to include pictures of common objects from the Snodgrass and Vanderwart (1980) collection. Stimuli were chosen on the basis of having an age of acquisition below 8 years and a name agreement level of over 65% in children aged 5–6 years (Snodgrass and Vanderwart, 1980; Cycowicz et al., 1997). Participants viewed a sequence of object pictures which appeared centrally on screen and were required to press a button for all Go pictures (manmade objects) and to withhold response for No-Go pictures (natural objects). The experimental block consisted of 100 trials (75 go trials and 25 no-go trials). A practice block of 20 trials with feedback was given prior to the experimental block. Stimuli appeared on screen for 2,000 ms with an inter-stimulus fixation for 1,000 ms. Stimuli were presented in the same pseudo-random order for each participant. The dependent measure on this task was the percentage commission errors committed. The Go No-Go paradigm of task significantly loads on to an inhibitory control factor (Archibald and Kerns, 1999), and is underpinned by frontal brain activation (Casey et al., 1997; Booth et al., 2005).

##### Phoneme Go No-Go task

Participants completed the phoneme Go No-Go task as a measure of inhibition. This task was an adapted version of the Go No-Go task (Brocki and Bohlin, 2004; McAuley and White, 2011) to include phoneme-picture information. Stimuli were selected from the Snodgrass and Vanderwart (1980) collection on the basis of picture name being monosyllabic or bi-syllabic, having an age of acquisition below 8 years and a name agreement level of over 65% in children aged 5–6 years (Snodgrass and Vanderwart, 1980; Cycowicz et al., 1997). Participants viewed a sequence of pictures which appeared centrally on screen and were required to press a button for Go stimuli (pictures beginning with a consonant) and to withhold response for No-Go stimuli (pictures beginning with a vowel). The experimental block consisted of 100 trials (75 Go trials and 25 No-Go trials). A practice block of 20 trials with feedback was given prior to experimental block. Stimuli appeared on screen for 2,000 ms with an inter-stimulus fixation for 1,000 ms. Stimuli were presented in the same pseudo-random order for each participant. The dependent measure on this task was the percentage commission errors committed. The Go No-Go paradigm of task significantly loads on to an inhibitory control factor (Archibald and Kerns, 1999), and is underpinned by frontal brain activation (Casey et al., 1997; Booth et al., 2005).

##### Sustained attention to response task (SART)

Participants completed the random SART task as a measure of inhibition (Robertson et al., 1997; Johnson et al., 2007). Participants viewed a random sequence of single digits (1–9) on screen and were instructed to respond to all digits (go trials) with a button press except 3 (no-go trial). The experimental block consisted of 225 trials. A practice block consisting of 18 trials with feedback was administered prior to the experimental block. Single digits (1–9) appeared on screen for 313 ms, followed by a response cue for 563 ms and a fixation cross for 563 ms. Participants were instructed to respond when the response cue was on screen. The dependent measure on this task was the percentage commission errors committed. The random SART places demands on inhibition (Johnson et al., 2007), is similar in task procedure to Go No-Go task which significantly loads on to inhibitory control (Archibald and Kerns, 1999) and is underpinned by frontal brain activation (Fassbender et al., 2004).

##### Inhibition composite

Inhibition Z-mean composite scores were calculated to provide a cleaner measure of inhibition by filtering out non-executive noise and to increase power due to sample size constraints (Snyder et al., 2015). Z-mean composite scores were created rather than Z-scores to account for the influence of number of tasks contributing to the composite score. Z-scores for errors and reaction time from the Picture Go No-Go, Phoneme Go No-Go, SART, and Stroop task were combined to create inhibition composite scores as follows:

$$\begin{array}{l} \text{Error composite}:\\ \left(\frac{ZPicGNGComm+ZPhonGNG+ZSARTComm+ZStroopError}{4}\right)\\ \text{Reaction time composite}:\\ \left(\frac{ZPicGNGRT+ZPhonGNGRT+ZSARTRT+ZStroopRT}{4}\right) \end{array}$$

#### Updating measures

##### Letter 2-back task

Participants completed the letter 2-back (Kane et al., 2007) task as a measure of updating working memory. Participants viewed a continuous stream of letters presented centrally on screen and were required to decide if the current letter on screen matched the letter presented 2 times ago. If the letters matched participants were instructed to press the green button on the response pad and if the letters did not match participants were instructed to press the red button on the response pad. The experimental block consisted of 96 trials. Stimuli were presented on screen for 1,000 ms with an inter-stimulus fixation for 100 ms. Participants completed a practice block of 7 trials with feedback given prior to the experimental block. The dependent measure in this task is the percentage errors. The 2-back task loads on to a working memory updating factor (Wilhelm et al., 2013), and is underpinned by frontal brain activation (Owen et al., 2005).

##### Picture 2-back task

Participants completed the picture 2-back task as a measure of updating. This task was modified (Beneventi et al., 2010) to include basic visual information. Stimuli were selected from the Snodgrass and Vanderwart (1980) collection on the basis of having an age of acquisition below 8 years and a name agreement level of over 65% in children aged 5–6 years (Snodgrass and Vanderwart, 1980; Cycowicz et al., 1997). Participants were presented with a continuous stream of pictures appearing centrally on screen and were required to decide if the current picture on screen matched the picture that was on screen 2 times ago. If the pictures matched, participants were instructed to press the green button on the response pad and if pictures did not match participants were instructed to press the red button on the response pad. The experimental block consisted of 100 trials (33 of which were target matches). Participants completed a practice block of 20 trials with feedback prior to the experimental block. Stimuli appeared on screen for 1,000 ms with an inter-stimulus fixation for 1,500 ms. The dependent measure in this task is the percentage errors. The 2-back task loads on to a working memory updating factor (Wilhelm et al., 2013) and is underpinned by frontal brain activation (Owen et al., 2005; Beneventi et al., 2010).

##### Phoneme 2-back task

Participants completed the phoneme 2-back task as a measure of updating. This task was a modified version of the phoneme updating task used by Beneventi et al. (2010). This task was adapted for English speaking participants and only the first phoneme 2-back condition is used in the current study. Stimuli were selected from the Snodgrass and Vanderwart (1980) collection on the basis of picture name being monosyllabic or bi-syllabic, having an age of acquisition below 8 years and a name agreement level of over 65% in children aged 5–6 years (Snodgrass and Vanderwart, 1980; Cycowicz et al., 1997). Participants viewed a continuous sequence of pictures presented centrally on screen and were required to decide if the first phoneme of the current picture on screen matched the first phoneme of the picture presented on screen two times ago. If the phonemes matched participants were instructed to press the green button on the response pad and if phonemes did not match participants were instructed to press the red button on the response pad. The experimental block consisted of 100 trials (33 of which were target matches). Participants completed a practice block of 20 trials with feedback prior to the experimental block. Stimuli appeared on screen for 1,000 ms with an inter-stimulus fixation for 1,500 ms. The dependent measure in this task is the percentage errors. The 2-back task loads on to a working memory updating factor (Wilhelm et al., 2013) and is underpinned by frontal brain activation (Owen et al., 2005; Beneventi et al., 2010).

##### Updating composite

Updating Z-mean composite scores were calculated to provide a cleaner measure of updating by filtering out any non-EF noise and to increase power due to sample size (Snyder et al., 2015). Z-mean composite scores were created rather than Z-scores to account for the influence of number of tasks contributing to the composite score. Z-scores for errors and reactions times for the Picture 2-back, Phoneme 2-back, and Letter 2-back tasks were combined to create updating composite scores expressed as:

$$\begin{array}{l} \text{Error composite}:\\ \left(\frac{ZPic2backerror+ZPhon2backerror+ZLett2backerror}{3}\right)\\ \text{Reaction time composite}:\\ \left(\frac{ZPic2backRt+ZPhon2backRT+ZLet2backRt}{3}\right) \end{array}$$

#### Switching measures

##### Number-letter switch task

Participants completed the number-letter switch task as a measure of switching ability. An adapted version of the number-letter task (Rogers and Monsell, 1995; Miyake et al., 2000) was used where switch is based on the colour of stimuli instead of the location of stimuli. Participants were presented with different number-letter pairs (e.g., 2A) centrally on screen and were required to decide on the number if green or to decide on the letter if red. If the number-letter pair appeared in red participants had to focus on the letter and decide if it was a consonant or a vowel. If the number-letter pair appeared in green participants had to focus on the number and decide if it was even or odd. In the first block of 20 trials the number-letter pair only appeared in red. In the second block of 20 trials the number-letter pair only appeared in green. In the third block of 116 trials the number-letter pair changed between red and green and participants were required to switch between processing number or letter- switch occurred on every 4th trial. Participants completed a practice block of 12 trials with feedback prior to each experimental block. Stimuli appeared on-screen for 5,000 ms with an inter-stimulus fixation for 150 ms. The switch cost in errors and reaction time for this task is the difference between trials that required a switch and trials which required no switch. The number-letter switch task loads onto a switching construct (Miyake et al., 2000; Collette et al., 2005), and is underpinned by frontal brain activation (Collette et al., 2005).

##### Phoneme switch task

Participants completed the phoneme switch task as a measure of switching ability. The number letter-task procedure (Rogers and Monsell, 1995; Miyake et al., 2000) was adapted to contain phoneme information. Stimuli for this task were pictures of common objects from the Snodgrass and Vanderwart (1980) collection on the basis of picture name being monosyllabic or bi-syllabic, having an age of acquisition below 8 years and a name agreement level of over 65% in children aged 5–6 years (Snodgrass and Vanderwart, 1980; Cycowicz et al., 1997). Participants viewed a different number of pictures (e.g. 2 apples, 1 star, 3 balloons) on screen in light (light red, green, or blue) or dark colours (dark red, green or blue). Participants were required to do one of two things depending on the first phoneme (letter sound) of the pictures. If the first phoneme was a consonant-sound, participants had to decide if the pictures were light or dark in colour. If the first phoneme was a vowel-sound, participants had to decide if the number of pictures was even or odd. In the first block of 20 trials only first phoneme consonant pictures appeared on screen. In the second block of 20 trials only first phoneme vowel pictures appeared on screen. In the third block of 116 trials the pictures changed between first phoneme consonant and vowel, and participants were required to switch between processing number or colour- switch occurred on every 4th trial. Participants completed a practice block of 12 trials with feedback prior to each experimental block. Stimuli appeared on screen for 5,000 ms with an inter-stimulus fixation for 150 ms. The switch cost in errors and reaction time for this task is the performance difference between trials that required a switch and trials which required no switch. A similar task the number-letter switch task loads onto switching construct (Miyake et al., 2000; Collette et al., 2005), and is underpinned by frontal brain activation (Collette et al., 2005).

##### Switching composite

Switching Z-mean composite scores were calculated to provide a cleaner measure of switching by filtering out any non-executive noise and to increase power due to sample size constraints (Snyder et al., 2015). Z-mean composite scores were created rather than Z-scores to account for the influence of number of tasks contributing to the composite score. Z-scores for errors and reaction time were combined from the Number-Letter and Phoneme switch tasks to create switching composite scores expressed as:

$$\begin{array}{l} \text{Error composite}:\\ \left(\frac{ZNumLettswitcherrorcost+ZPhonswitcherrorcost}{2}\right)\\ \text{Reaction time composite}:\\ \left(\frac{ZNumLettswitchRTcost+ZPhonswitchRTcost}{2}\right) \end{array}$$

#### Reading

##### Reading ability

Participants completed the Green word reading list from the Wide Range Achievement Test 4 (WRAT-4) (Wilkinson and Robertson, 2006) as a measure of reading ability. The word reading subtest from WRAT-4 requires participants to read from a list of 55 items increasing in difficulty. The assessment was discontinued if participants had 10 consecutive errors. The WRAT-4 word reading subtest demonstrates good test retest reliability (subtest = 0.86) and consistency (subtest = 0.87) (Wilkinson and Robertson, 2006).

## Results

### Data analysis

To explore executive function profile associated with dyslexia, ANOVA, and ANCOVA analyses were performed. To explore the predictive ability of executive function z-mean composite scores for dyslexia diagnosis logistic regressions and receiver operating characteristic (ROC) curve analyses was performed. To explore whether executive function z-mean composites are predictive of variance in reading multiple linear regression analysis was performed. Preliminary analyses were conducted to ensure that variables did not violate the assumptions of normality, homogeneity of variance, homogeneity of regression slopes, independence of errors, multicollinearity, linearity, and linearity of logit. The Stroop interference effect in errors and reaction time on the Picture 2-back task violated the assumption of normality, appropriate non-parametric analysis was employed for these variables. All assumptions were met for the executive function z-mean composite scores for linear and logistic regression analyses.

#### Descriptive statistics

Descriptive statistics and between group comparisons for the dyslexia and control group are summarised in Table 3.

**Table 3:** Descriptive statistics and between group differences for dyslexia and control participants.

	**Dyslexia**			**Control**			**ANOVA**			**ANCOVA**			
**Measure**	**N**	**Mean**	**SD**	**N**	**Mean**	**SD**	**F/U**	***Df***	**P**	**F**	***Df***	**P**	**D**
Stroop RT effect	27	170.86	88.03	29	157.18	81.15	0.366	1.54	0.548	0.061	1.53	0.807	0.16
Stroop Error Effect	27	5.44	4.36	29	4.17	6.59	U = 282	Z = − 1.8	0.071	–	–	–	0.23
Pic. GNG % Comm.	27	20.33	13.37	28	9.43	8.93	12.75	1.53	0.001**	8.50	1.52	0.005**	0.94
Pic. GNG RT	27	680.06	91.08	28	711.82	124.21	1.162	1.53	0.286	2.47	1.53	0.122	−0.29
Phon. GNG % Comm.	27	24.15	12.67	29	16.55	12.27	5.193	1.54	0.027*	2.59	1.53	0.114	0.61
Phon. GNG RT	27	926.63	167.41	29	990.67	199.35	1.68	1.54	0.200	2.63	1.53	0.111	−0.35
SART % Comm.	27	41.48	16.94	29	31.31	12.61	6.56	1.54	0.013*	5.91	1.53	0.019*	0.68
SART RT	27	487.95	56.05	29	474.46	52.16	0.870	1.54	0.355	0.35	1.53	0.554	0.25
Inhibition Error Comp.	27	0.135	0.499	29	–0.391	0.546	13.85	1.53	0.000**	9.29	1.52	0.004**	1.01
Inhibition RT Comp.	27	−0.140	0.512	28	–0.091	0.790	0.09	1.53	0.771	0.760	1.52	0.387	−0.07
Let. 2-back % Error	27	59.25	16.51	29	41.37	13.17	19.14	1.54	0.000**	15.41	1.53	0.000**	1.20
Let. 2-back RT	27	578.57	90.3	29	611.56	55.43	2.75	1.54	0.103	1.37	1.53	0.247	−0.44
Pic. 2-back % Error	27	47.22	18.74	29	34.64	13.23	7.72	1.54	0.007**	3.88	1.53	0.054	0.47
Pic. 2-back RT	27	624.01	81.23	29	616.75	58.62	U = 353	Z = −0.63	0.268	–	–	–	0.10
Phon. 2-back % Error	27	67.92	12.78	29	65.82	13.08	0.280	1.54	0.650	0.003	1.53	0.957	0.16
Phon. 2-back RT	27	610.34	88.64	29	650.57	74.46	3.40	1.54	0.071	1.71	1.53	0.197	−0.49
Updating Error. Comp	27	0.169	0.78	29	–0.462	0.679	9.22	1.54	0.004**	5.68	1.53	0.021*	0.86
Updating RT Comp.	27	0.068	0.78	29	0.307	0.589	1.68	1.54	0.20	0.559	1.53	0.458	−0.35
Num-Let SW Error Cost	27	3.33	4.65	29	2.00	4.22	1.266	1.54	0.265	1.13	1.53	0.293	0.30
Num-Let SW RT cost	27	511.80	395.82	29	690.18	336.02	3.321	1.54	0.074	1.16	1.53	0.286	−0.49
Phon. SW err. Cost	27	1.67	4.84	29	2.55	4.31	0.524	1.54	0.472	0.266	1.53	0.608	−0.19
Phon. SW RT Cost	27	490.92	533.30	29	749.95	558.58	3.14	1.54	0.082	2.07	1.53	0.156	−0.47
Switch Cost Error Comp.	27	0.036	0.826	29	–0.024	0.62	0.070	1.54	0.793	0.131	1.53	0.719	0.08
Switch Cost RT Comp.	26	−0.103	0.818	29	0.368	0.75	5.03	1.54	0.029*	2.55	1.53	0.116	−0.60
Proc. Speed (no. items)	27	7.96	2.05	29	9.31	2.49	4.48	1.54	0.032*	–	–	–	−0.59
Reading	27	34.85	8.17	29	50.59	7.48	56.60	1.54	0.000**	47.10	1.53	0.000**	−2.01

Stroop RT, Stroop effect in reaction time; Stroop err, Stroop effect in error; GNG, GoNoGo; Comm, commission errors; Comp, composite score; RT, reaction time; Num-Let SW error, Number-Letter switch cost in errors; Num-Let SW RT, Number-Letter switch cost in reaction time; Phon SW err, Phoneme switch cost in errors; Phon SW RT, phoneme switch cost in reaction time; Proc. Speed, processing speed. P < 0.05*, P < 0.01**.

#### Executive function profile associated with dyslexia

##### Inhibition

**Figure 1:**
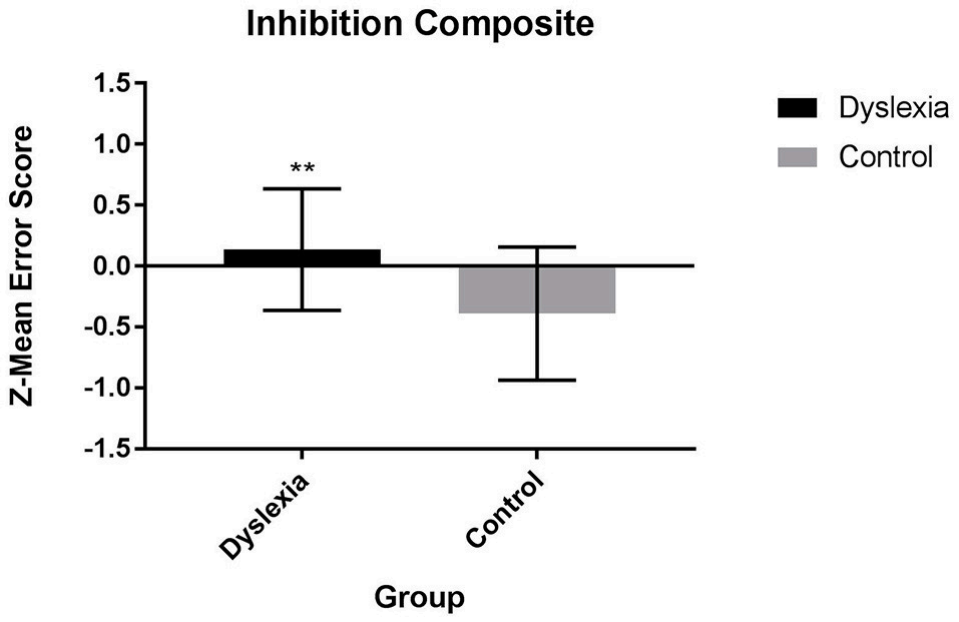
Inhibition Z-mean error composite scores for dyslexia and control participants.

Results from separate 2 (group: dyslexia, control) x 1 (inhibition measure: composite, task-level) ANOVAs indicate that dyslexia is associated with a significant inhibition impairment. At the composite level, dyslexia is characterized by a significantly higher inhibition z-mean error score than control participants [*F*_(1, 53)_ = 13.85, *p* = 0.000, Cohen's d = 1.01; Figure 1]. At the individual task-level, dyslexia is associated with significantly more commission errors than control participants during the Picture Go No-Go [*F*_(1,,53)_ = 12.75, *p* = 0.001, Cohen's d = 0.94], Phoneme Go No-Go [*F*_(1, 54)_ = 5.19, *p* = 0.027, Cohen's *d* = 0.61), and SART tasks [*F*_(1, 54)_ = 6.56, *p* = 0.013, Cohen's *d* = 0.68].

**Figure 2:**
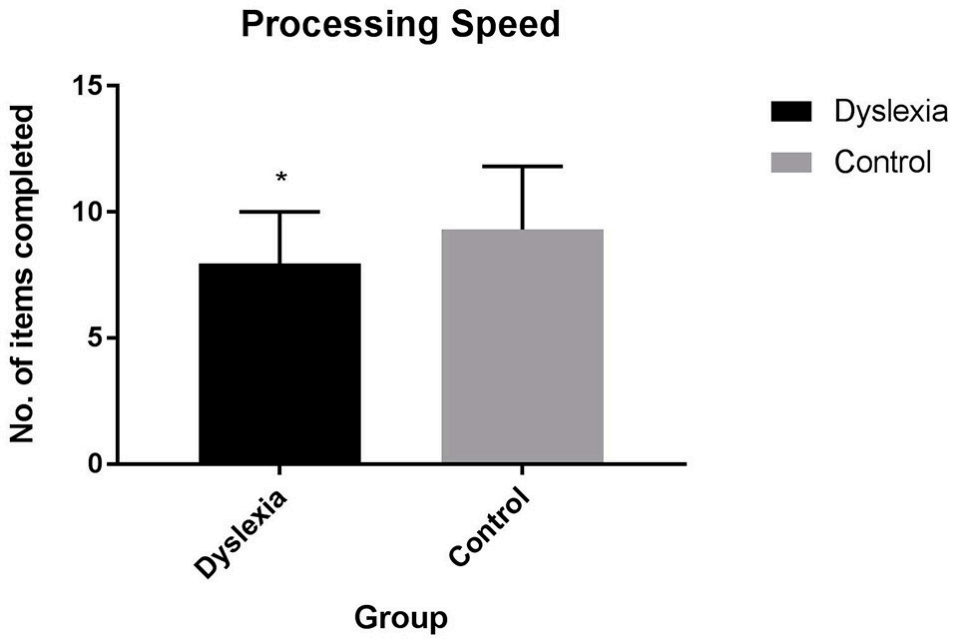
Processing speed scores for dyslexia and control participants.

Dyslexia is also associated with a processing speed impairment [*F*_(1, 54)_ = 4.84, *p* < 0.05, Cohen's *d* = 0.60; Figure 2]. After controlling for individual variation in processing speed, significant differences in the inhibition z-mean error score [*F*_(1, 52)_ = 9.29, *p* = 0.004], commission errors during the Picture Go No-Go task [*F*_(1, 52)_ = 8.50, *p* = 0.005] and commission errors during the SART task [*F*_(1, 53)_ = 5.91, *p* = 0.019] remain. Group differences in commission errors on the Phoneme Go No-Go [*F*_(1, 53)_ = 2.59, *p* = 0.114] task are no longer significant after controlling for individual variation in processing speed.

No significant group differences were observed for the inhibition z-mean reaction time composite score, Stroop task (Stroop effect in reaction time or error), or reaction time during the Picture Go No-Go, Phoneme Go No-Go and SART tasks, before or after controlling for individual variation in processing speed (see Table 3).

##### Updating

**Figure 3:**
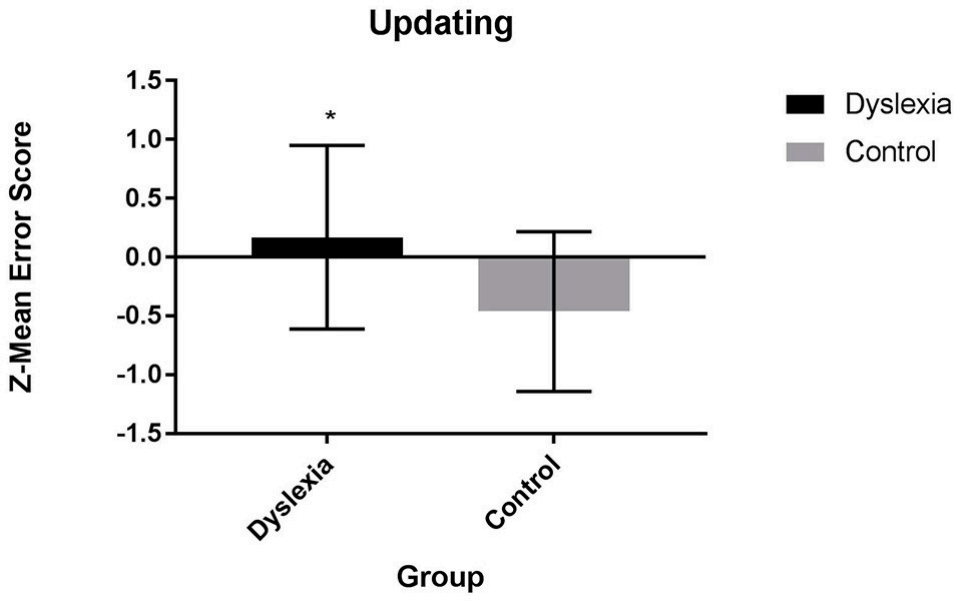
Updating Z-mean error composite scores for dyslexia and control participants.

Results from separate 2 (group: dyslexia, control) x 1 (updating measure: composite, task-level) ANOVAs indicate that dyslexia is associated with a significant updating impairment. At the composite level, dyslexia is characterized by a significantly higher updating z-mean error score than control participants [*F*_(1, 54)_ = 19.14, *p* = 0.004, Cohen's *d* = 0.86, Figure 3]. At the individual task-level, dyslexia is associated with significantly more errors during the Letter 2-back [*F*_(1, 54)_ = 19.14, *p* = 0.000, Cohen's *d* = 1.20] and Picture 2-back [*F*_(1, 54)_ = 7.72, *p* = 0.007, Cohen's *d* = 0.47) tasks.

After controlling for individual variation in processing speed, significant differences for the updating z-mean error composite score [*F*_(1, 53)_ = 5.68, *p* = 0.021] and errors during the Letter 2-back task [*F*_(1, 53)_ = 15.41, *p* = 0.000] remained. Group differences in errors on the Picture 2-back task are no longer significant [*F*_(1, 53)_ = 3.88, *p* = 0.054) after controlling for individual variation in processing speed.

No significant differences were observed for the updating z-mean reaction time composite score, Phoneme Go No-Go task (error rate or reaction time), or reaction time during the Letter 2-back and Picture 2-back tasks, before or after controlling for individual variation in processing speed (see Table 3).

##### Switching

Results from separate 2 (group: dyslexia, control) x 1 (switching measure: composite, task level) ANOVAs indicate that dyslexia is associated with a switching strength. At the composite level, dyslexia is characterized by a significantly lower switching z-mean reaction time cost score than control participants [*F*_(1, 54)_ = 5.03, *p* = 0.029, Cohen's *d* = −0.60].

After controlling for individual variation in processing speed, significant differences for the switching z-mean reaction time cost score are no longer significant [*F*_(1, 53)_ = 2.55, *p* = 0.116].

**Figure 4:**
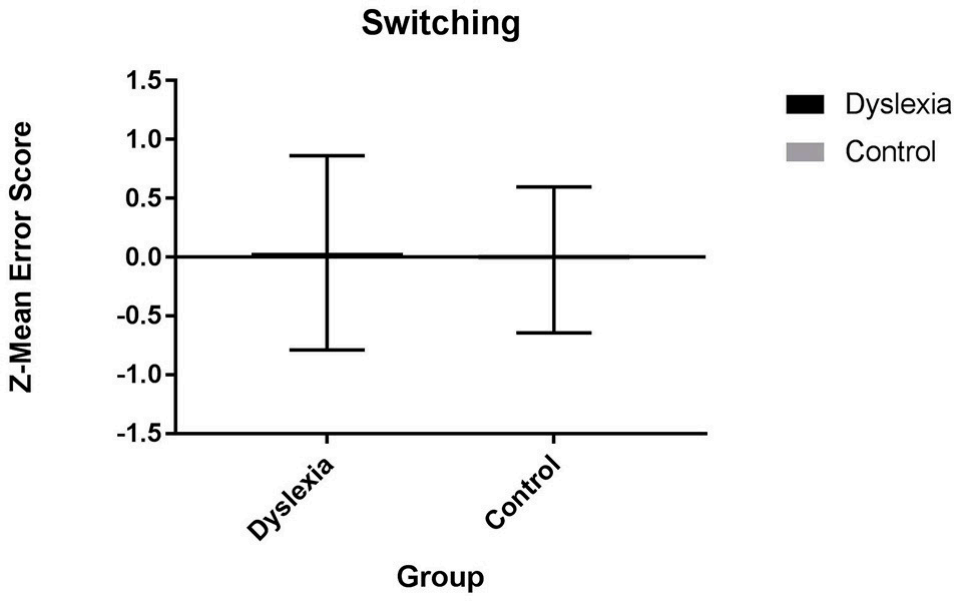
Switching Z-mean error composite scores for dyslexia and control participants.

No significant differences were observed for the switching z-mean error composite score (see Figure 4), Number-Letter Switch task (reaction time cost or error cost) or the Phoneme Switching task (reaction time cost or error cost), before or after controlling for individual variation in processing speed (see Table 3).

#### Predicting dyslexia likelihood

Results from the binary logistic regression are summarised in Table 4. At step 1, processing speed only was entered into the model to control for its influence on executive function. At step 2, in addition to processing speed, inhibition, updating and switching z-mean error composite scores were entered into the model respectively to reflect the pattern of impaired and unimpaired processes associated with dyslexia.

**Table 4:** Binary logistic regression with executive function error composite scores.

	**Binary logistic regression (Dyslexia vs. Control)**			
	**β (SE)**	**Exp (B)**	**95% CI**	**−2Log Likelihood**
**Step 1**				70.94
Constant	2.41 (1.18)	11.16		
Processing Speed	−0.282 (0.131)*	0.754	0.584–0.975	
**Step 2**				55.45
Constant	0.841 (1.42)	2.32		
Processing Speed	−0.051 (0.168)	0.950	0.684–1.32	
Inhibition	1.83 (0.688)*	6.23	1.62–24.00	
Updating	1.28 (0.565)*	3.61	1.19–10.92	
Switching	0.031 (0.468)	1.03	0.413–2.58	

**Step 1:** R^2^ = 0.092 (Cox & Snell), 122 (Nagelkerke), Model X^2^(1) = 5.29, p < 0.05. **Step 2:** R^2^ = 0.315 (Cox & Snell), 0.42 (Nagelkerke), Model X^2^(4) = 20.77, p < 0.001. Hosmer and Lemeshow (Step 1) X^2^(6) = 10.13, p = 0.119, (Step 2) X^2^(7) = 8.19, p = 0.316 indicates good model fit. P < 0.05*, P < 0.01**.

Step 1 (processing speed): demonstrated a trend for predicting dyslexia, the chi square [X^2^(1) = 5.29, *p* = 0.032] and−2Log Likelihood (70.94) statistics demonstrate good model fit. Model 1 correctly classified 65.5% of participants according to presence/absence of dyslexia diagnosis: sensitivity 59.3% (true-positive) and specificity 71.4% (true-negative).

The addition of the inhibition, updating and switching composite scores at step 2, significantly improved model fit [Chi square: Model X^2^(3) = 15.49, *p* = 0.001; −2Log Likelihood: 55.45; ${\text{R}}_{\text{cs}}^{2}$ = 0.315; ${\text{R}}_{\text{N}=0.}^{2}$42]. This model correctly classified 78.2% of participants according to presence/absence of dyslexia diagnosis: sensitivity 81.5% (true-positive) and specificity 75% (true-negative). As outlined in Table 4, this model suggests that when accounting for low-level processing speed only inhibition [Wald: X^2^(1) = 7.06, *p* = 0.008] and updating composite scores [Wald: X^2^(1) = 5.17, *p* = 0.023] predict dyslexia. The b-values reflect that for every for one-unit change in inhibition score (errors) there is a corresponding 1.83-unit change in the logit of the outcome variable, while for every one-unit change in updating score (errors) there is a 1.28-unit change in the logit of the outcome variable. The proportionate odds values [Exp (B)] are >1 for both predictors suggesting that as error score on each predictor increases the likelihood of the outcome occurring (dyslexia diagnosis) increases.

**Figure 5:**
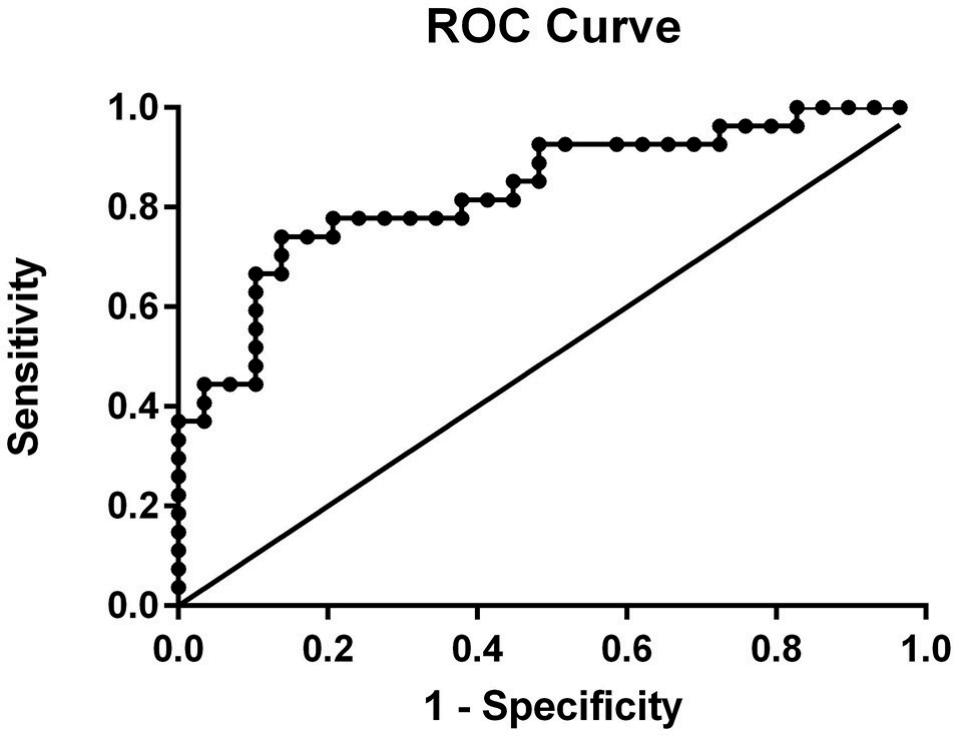
ROC curve of inhibition and updating Z-mean error scores for predicting dyslexia likelihood.

ROC curve analysis (see Figure 5) indicates that the executive function predictive model (inhibition and updating) is a good fit with an area under the curve (AUC) of.835 (95% CI:0.727–0.942, *p* = 0.000). A randomly selected participant with dyslexia will have a higher error rate on inhibition and updating composites than a randomly selected control participant approximately 83.5% of the time. According to Swets (1988), criteria for diagnostic accuracy (poor:0.5–0.7, moderate:0.7–0.9, high:0.9–1.0), inhibition and updating composites demonstrate moderate accuracy in predicting dyslexia diagnosis.

#### Predicting reading ability

Hierarchical multiple linear regression is explored here with processing speed entered at step 1 and inhibition, updating, and switching error composites scores entered respectively at step 2 to address whether core executive functions are predictive of reading ability (see Table 5 for results). Hierarchical multiple linear regression was explored within dyslexia alone and control alone (see Tables 6, 7 for summary of results). It should be noted that overall each model was non-significant (dyslexia: *R*^2^ = 0.275, *p* = 0.07; control: *R*^2^ = 0.287, *p* = 0.09). However, exploring the predictive relationship between cognitive processes and behavioural outcomes separately in clinical and non-clinical groups has recently been criticized as it does not include the full dimension of variability from typical to atypical (Cuthbert, 2014). For this reason, we sought to explore the predictive relationship between core executive functions and reading ability to understand how it related to the full dimension of reading ability.

**Table 5:** Linear regression model with executive function error composites predicting reading ability across groups.

	**Reading Ability Across Groups**				
	**B**	**SEB**	**β**	**F/T-Value**	**P**
**Step 1**				6.83	0.012*
Constant	29.30	5.43			
Processing speed	1.58	0.61	0.338	2.61	0.012*
**Step 2**				10.61	0.000**
Constant	39.43	4.79			
Processing speed	−0.19	0.556	0.041	0.341	0.734
Inhibition	−10.03	2.14	−0.527	−4.68	0.000**
Updating	−4.31	1.65	−0.307	−0.262	0.012*
Switching	−1.35	1.7	−0.088	−0.799	0.428

**Step 1:** R^2^ = 0.114; **Step 2:** R^2^ = 0.459.

* p < .05,

** *p < 0.01*.

**Table 6:** Linear regression model with executive function error composites predicting reading ability dyslexia alone.

	**Reading ability across groups**				
	**B**	**SEB**	**β**	**F/T-Value**	**P**
**Step 1**				0.201	0.658
Constant	32.01	6.53			
Processing speed	0.357	0.795	0.089	0.448	0.658
**Step 2**				2.71	0.07
Constant	34.11	6.32			
Processing speed	0.249	0.759	0.062	0.329	0.745
Inhibition	−7.56	2.99	−0.461	−2.52	0.019*
Updating	−0.846	2.08	−0.081	−0.407	0.688
Switching	−2.036	1.89	−0.233	−1.22	0.235

**Step 1:** R^2^ = 0.008; **Step 2:** R^2^ = 0.275.

* p < 0.05,

** *p < 0.01*.

**Table 7:** Linear regression model with executive function error composites predicting reading ability control alone.

	**Reading ability across groups**				
	**B**	**SEB**	**β**	**F/T-Value**	**P**
**Step 1**				2.85	0.103
Constant	41.51	5.48			
Processing speed	0.954	0.565	0.314	1.67	0.103
**Step 2**				2.31	0.088
Constant	45.52	5.54			
Processing speed	0.091	0.650	0.030	0.140	0.890
Inhibition	−5.60	2.88	−0.403	−1.94	0.064
Updating	−4.16	2.17	−0.373	−1.91	0.069
Switching	−0.195	2.29	0.016	−0.085	0.933

**Step 1:** R^2^ = 0.099; **Step 2:** R^2^ = 0.287. *p < 0.05, **p < 0.01.

Step 1 (processing speed): significantly predicted 11.4% of the variance in reading ability across groups. Step 2 (processing speed and executive function): Adding executive function composite scores to the model significantly improved the predictive ability (45.9%) and explained an additional 34.5% of the variance in reading ability [R^2^change = 0.345, *F*_(3, 54)_ = 25.98, *p* = 0.000]. As outlined in Table 5, the results suggest that after controlling for processing speed abilities inhibition and updating significantly predict reading ability. Beta values for inhibition and updating reflect a 0.527 and 0.307 decrease in reading ability score for every 1SD increase in executive function composite error respectively. This suggests that inhibition and updating can predict variance in reading abilities across a trajectory from typical-atypical reading.

#### Summary of results

Dyslexia is associated with inhibition and updating impairments while controlling for individual variation in processing speed impairments. Inhibition and updating are clinically relevant for predicting dyslexia likelihood and reading ability while controlling for individual variation in processing speed.

## Discussion

From previous research, the core executive function profile (strengths and impairments in inhibition, updating, and switching) associated with dyslexia alone is unclear. Inconsistent impairments are found across a range of executive measures in dyslexia. In addition, there are inconsistencies regarding which exact aspects of executive function are predictive of dyslexia likelihood and reading ability. Potential reasons for inconsistent findings across the literature include discrepancies with group classification, theoretical approach to profiling, task impurity issues, and a lack of control for the confounding influence of processing speed on executive function. These issues make it increasingly difficult to infer the core executive function profile associated with dyslexia and whether core executive functions are clinically relevant for predicting dyslexia diagnosis and variance in reading ability. This study contributed to existing literature on executive functions in dyslexia by employing sensitive measures of each core executive construct (z-mean composites) within the 3-factor model of executive function (Miyake et al., 2000; Miyake and Friedman, 2012; Snyder et al., 2015) while controlling for individual variation in processing speed, in a homogenous sample of children with dyslexia (clinical diagnosis, screened for elevated ADHD with a standardised measure). Findings suggest that dyslexia is associated with inhibition and updating, but not switching impairments at the z-mean composite level, whilst controlling for individual variation in processing speed. Inhibition and updating, but not switching, were also predictive of dyslexia likelihood and reading ability, whilst controlling for individual variation in processing speed.

The model for predicting dyslexia likelihood developed in this study demonstrated that inhibition and updating error composites significantly predict likelihood of dyslexia (sensitivity: 81.5%, specificity: 75%) with moderate diagnostic accuracy (0.835) according to Swets (1988) criteria (poor:0.5–0.7; moderate:0.7–0.9, high:0.9–1.0). The accuracy rate suggests that a randomly selected participant with dyslexia will have a higher error rate on inhibition and updating z-mean error composites than a randomly selected control participant approximately 83.5% of the time. These findings suggest that inhibition and updating abilities not only differentiate dyslexia from control participants but are capable of discriminating dyslexia from control participants.

The predictive ability of inhibition and updating for dyslexia likelihood found in this study is consistent with the work of Booth et al. (2014), which found that a model including inhibition and working memory abilities predict dyslexia likelihood. Booth et al. (2014) found that a model including a non-verbal working memory task and an inhibition composite score (comprised of Stroop task and Number-Detection task performance) correctly classified 78% of participants according to absence/presence of dyslexia (sensitivity: 86%; specificity: 65%). However, in their model only the inhibition composite score discriminated between dyslexia and control participants (Booth et al., 2014). Although, our findings are similar to Booth et al. (2014), their study did not include measures of switching, control for processing speed and included 9 dyslexia participants with elevated ADHD. The findings from our dyslexia predictive model are inconsistent with the work of Moura et al. (2015), which found that switching abilities predict dyslexia likelihood. Moura et al. (2015) found that switching, as measured with the Trail Making Task, significantly predicts dyslexia likelihood with moderate diagnostic accuracy (0.73), and correctly classifies 71.7% of participants according to absence/presence of dyslexia (sensitivity: 69.4%; specificity: 74%). However, their study did not use a screening tool to remove potential undetected ADHD, include measures of the other core executive functions (inhibition and updating) and did not control for processing speed. The model developed in the present study demonstrates higher diagnostic accuracy (0.835) and correctly classifies a higher proportion of participants (78.2%) than both previous studies (Booth et al., 2014; Moura et al., 2015). To our knowledge, the present study is the first to explore the ability of all three core executive functions for predicting dyslexia likelihood while controlling for processing speed. Although our model found that processing speed predicts dyslexia likelihood, after the executive function error composites were included, the predictive relationship between processing speed and dyslexia likelihood was no longer significant. The only core executive functions predictive of dyslexia likelihood were inhibition and updating, suggesting that these abilities can discriminate dyslexia from control participants.

Inhibition and updating composites also significant predicted reading ability. The model for predicting reading ability developed in this study explained 45.9% of the variance in reading ability. The initial model including only processing speed at step 1 demonstrated a trend for predicting variance in reading ability (11.4%), however executive function z-mean error composites significantly improved the model's predictive ability, explaining an additional 34.5% of variance in reading ability. Processing speed was no longer significant after executive functions were entered and the model suggested that inhibition and updating were the only significant core executive predictors of reading. The relationship was such that those with higher errors on inhibition and updating z-mean composites had significantly poorer reading ability.

The predictive relationship of inhibition and updating for reading ability is consistent with previous work finding that inhibition and working memory combined are predictive of reading in typical samples (Welsh et al., 2010; Arrington et al., 2014) and that working memory and inhibition are predictive of the severity of reading impairment expressed in dyslexia (Wang and Yang, 2015). Arrington et al. (2014) found that working memory (Digit Span Backward task) and response inhibition (Stop Signal task) predicted word reading ability. However, Wang and Yang (2015) found that working memory (sentence span) and a cognitive inhibition composite score (comprised of Stroop and Group Embedded Figures task performance), but not a behavioural inhibition composite score (comprised of Go No-Go and Stop signal task performance), predicted reading ability in dyslexia. The findings of the present study are more similar to those of Arrington et al. (2014), as our inhibition composite predicting reading ability was more heavily weighted on response inhibition (Picture Go No-Go, Phoneme Go No-Go, and SART tasks) than cognitive inhibition (Stroop task) which did not differentiate participants at the task level. However, both studies did not include measures of switching and updating, or control for the influence of processing speed on executive performance. Christopher et al. (2012) found that working memory (sentence span, digit span, counting span) and processing speed (perceptual speed, identical pictures), but not inhibition (continuous performance, stop signal tasks) latent factors predict reading ability. The predictive relationship between processing speed and reading is also found in other studies (McGrath et al., 2011; Peterson et al., 2016). Yet, our findings suggest that after including core executive functions in the reading model processing speed is no longer a significant predictor, while inhibition and updating are the only significant predictors of reading ability.

Poor performance on inhibition and updating composites was associated with poor reading ability. This suggests that the core executive functions of inhibition and updating support word reading ability and when disrupted as is the case in dyslexia contribute to reading impairment. As previously discussed, efficient reading requires the coordination of multiple processes such as focusing of attention on visual information, decoding visual information into speech sounds, maintaining, and updating speech sounds in working memory, combining speech sounds, matching combinations of speech sounds with stored words, deriving semantic meaning for comprehension, and moving onto the next word to start this process again. Our findings suggest that inhibition can contribute to this process, children with dyslexia experience inhibition difficulties which may result in a difficulty suppressing irrelevant information and protecting the contents of working memory. As suggested by Arrington et al. (2014) response inhibition in particular, may be an important gating function preventing activation of similar words with spelling-sound mappings in working memory. This constraint may also result in further difficulty gating external information such as classroom noise from working memory while reading.

Children with dyslexia also experience working memory updating impairments, this difficulty may result in reading difficulty due to an inability/reduced capacity to hold and update speech sounds in working memory during ongoing decoding. Switching was unimpaired in dyslexia and did not predict reading, therefore children with dyslexia do not appear to struggle with the rapid alteration between different demands.

The findings from the present study suggest that dyslexia is associated with impaired inhibition and updating while controlling for processing speed. These findings are consistent with previous research documenting impaired inhibition (Helland and Asbjørnsen, 2000; Willcutt et al., 2005, 2007; de Jong et al., 2009; De Lima et al., 2012; Booth et al., 2014; Wang and Yang, 2015), impaired updating/working memory (Beneventi et al., 2010; Booth et al., 2014; Wang and Yang, 2015), and unimpaired switching in dyslexia (Reiter et al., 2005; Willcutt et al., 2005; Bental and Tirosh, 2007; Menghini et al., 2010; Moura et al., 2015). However, all of these studies explored group differences at the individual task level and not at the composite level. To our knowledge this is the first study to explore all three core executive functions (inhibition, updating, and switching) within the same study in dyslexia with more sensitive z-mean measures while controlling for individual differences in processing speed.

Only one study thus far has controlled for the confounding influence of processing speed on the performance profile of executive functions associated with dyslexia (Peng et al., 2013). Peng et al. (2013) found updating and inhibition impairments in dyslexia, yet when they controlled for general processing speed impairments, updating and inhibition impairments no longer reached significance. The findings from this study are inconsistent with Peng et al. (2013), suggesting that inhibition and updating impairments remain in dyslexia even while controlling for the confounding influence of processing speed. For inhibition, impairments remained in dyslexia at the composite level and individual task level (Picture Go No-Go, SART task) while controlling for processing speed. However, impairments on the Phoneme Go No-Go task in dyslexia were no longer significant after accounting for individual differences in processing speed. For updating, impairments remained at the composite level and individual task level (Letter 2-back task) while controlling for processing speed. However, impairments on the Picture 2-back task were no longer significant after controlling for processing speed. For switching, a significant strength on the z-mean reaction time switch cost score was found, however, this was no longer significant after controlling for speed.

The pattern of findings suggest that processing speed may mediate some performance in the core executive functions of inhibition and updating at the task level, and, switching at the composite level. Consistent with previous work, this study found a processing speed impairment in dyslexia (Willcutt et al., 2005; McGrath et al., 2011). Despite accounting for some variability in performance, inhibition and updating impairments remain in dyslexia while controlling for processing speed. These findings relate to the previous work conducted by Huizinga et al. (2006), who found that inhibition and switching tasks load onto a processing speed factor. This may explain why switching strengths were no longer significant in dyslexia after controlling for individual variation in processing speed. However, we also found that processing speed can account for impairments on some inhibition and updating tasks in dyslexia also. These findings support previous work that processing speed mediates some executive function performance (Span et al., 2004). Despite accounting for some performance in executive function tasks in dyslexia, we found that processing speed does not account for inhibition and updating impairments in dyslexia. Suggesting inhibition and updating impairments in dyslexia are not accounted for by individual variation in processing speed as suggested by Peng et al. (2013).

However, issues flagged in prior work relating to measurement of core executive functions make it difficult to relate our specific findings to previous work (Miyake and Friedman, 2012; Goschke, 2014; Snyder et al., 2015; Friedman and Miyake, 2016). By employing more sensitive z-mean executive function composite scores to reduce non-executive noise and isolate core executive processes (Snyder et al., 2015), this study found for the first time that inhibition and updating impairments are associated with dyslexia while controlling for processing speed and that inhibition and updating abilities are predictive of dyslexia likelihood and reading ability across the spectrum of typical to atypical reading while controlling for processing speed. We would argue that executive function, particularly inhibition, may underlie the severity of reading impairments in dyslexia.

By exploring the executive function profile associated with dyslexia using Miyake and Friedman's (2012) three factor model, it is apparent that inhibition (common executive function) may be the central executive function impairment associated with dyslexia. Inhibition was the most severe impairment associated with dyslexia; and within predictive models of both dyslexia likelihood and reading ability, it was the most significant and heavily weighted predictor. This ‘common executive’ impairment may lead to impaired updating and unimpaired switching due to shared variance (Friedman et al., 2006, 2007, 2008; Miyake and Friedman, 2012) and antagonistic relationships such as performance trade-offs between inhibition and switching (Goschke, 2000; Gruber and Goschke, 2004; Blackwell et al., 2014). For instance, inhibition facilitates focus by shielding information from irrelevant distractors in a top down manner (provides stability), while switching requires interference from distractors to consider alternative options and to flexibly adapt to changing demands (mental flexibility; Gruber and Goschke, 2004). This may be the reason why the present study found impaired inhibition and updating, and spared switching abilities associated with dyslexia. Operationally defining and measuring executive function within the 3-factor latent model (Miyake and Friedman, 2012) allows us to see that executive functions may operate in a strengths and impairments manner (Snyder et al., 2015).

Overall results from this study suggest that dyslexia is associated with inhibition and updating impairments, which are predictive of disorder likelihood and variability in reading, even when controlling for processing speed. These findings suggest that inhibition impairments are implicated in dyslexia and predict individual differences in reading ability.

This study is not without limitations. Although our measure of processing speed loads highly on processing speed in factor analytic studies (Keith et al., 2006; Watkins et al., 2006; Bodin et al., 2009), some authors report that this task is correlated with inhibition and predicts variance in working memory (Cepeda et al., 2013). Cepeda et al. (2013) caution against using processing speed measures which are correlated with executive functions as they may overestimate the role of processing speed in executive processes. As such, a possible limitation of our study is that by using the coding task as a measure of processing speed we removed important executive associated variance from our measures. Therefore, our study may underestimate the degree to which executive function is impaired in dyslexia. Another limitation is that the limited number of switch trials in the switching tasks (e.g., a switch occurred on every 4th switch trial) mat have resulted in unreliable data for this measure. In addition, although this study attempted to derive purer measures of each core executive construct by calculating z-mean composite scores from performance across multiple measures, non-executive similarities in the measures used may contribute variance to the composite score. For example, this study employed three 2-back tasks which included different stimuli (e.g., picture, phoneme, letter), all of these tasks required a button press and visual identification of stimuli. As such, this variance may be contributing to the composite score. In addition, the majority of tasks contributing to the inhibition composite scores were Go No-Go style tasks, meaning that this measure is more heavily weighted on response inhibition rather than interference control as measured by the Stroop task.

Future research should explore the core executive functions in dyslexia while controlling for processing speed with latent analysis techniques and structural equation modelling, and explore whether inhibition training can improve core executive functions and reading ability in children with dyslexia.

## Author contributions

CD and LB ideated the project and designed the protocol. CD created the EPrime measures, led the data acquisition, data analysis, and wrote the initial version of the paper. LB contributed to the interpretation of the data, revised the paper for intellectual content, reviewed, and refined the paper. AS and RR reviewed and refined the paper. All authors agree to be accountable for all aspects of the work.

### Conflict of interest statement

The authors declare that the research was conducted in the absence of any commercial or financial relationships that could be construed as a potential conflict of interest.
